# The Study Team for Early Life Asthma Research (STELAR) consortium ‘Asthma e-lab’: team science bringing data, methods and investigators together

**DOI:** 10.1136/thoraxjnl-2015-206781

**Published:** 2015-03-24

**Authors:** Adnan Custovic, John Ainsworth, Hasan Arshad, Christopher Bishop, Iain Buchan, Paul Cullinan, Graham Devereux, John Henderson, John Holloway, Graham Roberts, Steve Turner, Ashley Woodcock, Angela Simpson

**Affiliations:** 1 Centre for Respiratory Medicine and Allergy, Institute of Inflammation and Repair, University of Manchester and University Hospital of South Manchester, Manchester, UK; 2 Centre for Health Informatics, Institute of Population Health, University of Manchester, Manchester, UK; 3 David Hide Asthma and Allergy Research Centre, St Mary's Hospital, Newport, Isle of Wight, UK; 4 Clinical and Experimental Sciences Academic Unit, University of Southampton Faculty of Medicine, Southampton, UK; 5 NIHR Respiratory Biomedical Research Unit, University Hospital Southampton NHS Foundation Trust, Southampton, UK; 6 Microsoft Research Cambridge, Cambridge, UK; 7 Imperial College, London, UK; 8 Child Health, University of Aberdeen, Aberdeen, UK; 9 School of Social and Community Medicine, University of Bristol, Bristol, UK; 10 Human Developmental and Health Academic Unit, University of Southampton Faculty of Medicine, Southampton, UK

**Keywords:** Asthma, Asthma Epidemiology, Paediatric asthma

## Abstract

We created Asthma e-Lab, a secure web-based research environment to support consistent recording, description and sharing of data, computational/statistical methods and emerging findings across the five UK birth cohorts. The e-Lab serves as a data repository for our unified dataset and provides the computational resources and a scientific social network to support collaborative research. All activities are transparent, and emerging findings are shared via the e-Lab, linked to explanations of analytical methods, thus enabling knowledge transfer. eLab facilitates the iterative interdisciplinary dialogue between clinicians, statisticians, computer scientists, mathematicians, geneticists and basic scientists, capturing collective thought behind the interpretations of findings.

Asthma epidemiology is reaching the limit of what can be achieved through conventional hypothesis-driven research, due to factors that include residual confounding, multiple influences of modest effect size and lack of statistical power to detect interactions between factors in complex datasets. Evidence is mounting that asthma is not a single disease, but a collection of several diseases, each with unique pathophysiology, and environmental and genetic associates (often referred to as ‘asthma endotypes’).^1^ Some reports have proposed abolishing the term ‘asthma’.^2^ This conceptual framework of ‘asthma syndrome’ may also explain why the responses to currently available treatments vary considerably: if asthma is an umbrella diagnosis that comprises multiple diseases with distinct mechanisms, then it is unlikely that these different diseases would always respond equally to same therapeutic agents. In this context, ‘asthma phenotype’ can be viewed as an observable characteristic that can be shared between several diseases (‘endotypes’) within the asthma syndrome, while ‘asthma endotype’ is a hypothetical construct that has a tangible value in helping us to better understand the underlying pathophysiological mechanisms of asthma disease-related states and may help identify more effective personalised treatment strategies. However, unless better ways are found to distinguish between different endotypes, it will be difficult to discover their underlying pathophysiological processes or identify novel therapeutic targets for stratified treatment, as any signal will be diluted by phenotypical heterogeneity.^1^

Asthma usually starts early in life, and may progress, remit or relapse over time. Temporal analysis is therefore crucial for distinguishing between different subtypes of asthma, and the population-based birth cohort (which overcomes recall bias and permits longitudinal phenotyping) is the preferred study design for investigating this kind of disease development.^3^ Birth cohorts collectively may contain fundamentally important data to elucidate true endotypes of childhood asthma. A major challenge for epidemiology is how best to use the vast amount of data generated in multiple birth cohorts, how to integrate different scales of data (from molecular-level, genetic and epigenetic, to population-level variables and environmental exposures) and different levels of directness of measurement of the many variables of interest. Pooling resources and multidisciplinary expertise from different centres to maximise the potential of gathered data may facilitate the improvement of health outcomes (http://www.farrinstitute.org). Ideally, researchers would use all available data (eg, multiple questions, directly observed exposure and outcome measures and laboratory readouts at multiple time points) to identify latent structure within the data that may arise from different pathophysiological processes underlying endotypes. Such models need to be tailored to individual datasets and be able to scale up to large volumes of data, taking into account the time course of developmental profiles at individual level. Rather than using a ‘black box’ or ‘data-mining’ approach, this process should be informed by the current and future biological and clinical knowledge about asthma. For example, pathological and physiological processes can be mapped out in computable forms using a technique called *graphical modelling*, which creates *annotated dependency graphs* of the underlying biological processes. To do this effectively, it is essential to integrate three key ingredients: (1) data, (2) models/methods that can be tailored in full to the problem space and (3) human expertise to tailor models/methods and make sense of results in context. Bringing the data, methods and investigators together on-line is one of the key objectives of the Study Team for Early Life Asthma Research (STELAR) consortium.

## STELAR consortium

In 2002, Asthma UK commissioned the creation of a database of the UK-based birth cohorts focusing on asthma. Recognising their commonality of purpose, the investigators from five birth cohorts (Avon Longitudinal Study of Parents and Children, Ashford and Isle of Wight cohorts, Manchester Asthma and Allergy Study and the Aberdeen Study of Eczema and Asthma To Observe the Effects of Nutrition) created the *STELAR network*. Individually, each cohort has great depth in terms of exceptionally well-characterised subjects, a wealth of environmental exposure measures and genetic and other biological data. Collectively, the STELAR network gains power from this breadth (data are available on >14 000 children with many repeat measures over time). A further strength is derived from the collaborative agreement to introduce harmonised protocols to develop common outcomes, which facilitates data pooling.

*The STELAR consortium* is truly multidisciplinary, combining expertise in birth cohorts (the *STELAR network*), with molecular genetics, epidemiologically oriented health informatics research and statistical machine learning, to capitalise on the rich resource available within the cohorts.

### STELAR Asthma e-Lab: a secure web-based resource for managing the data, metadata and collaborative analysis

**Figure 1:**
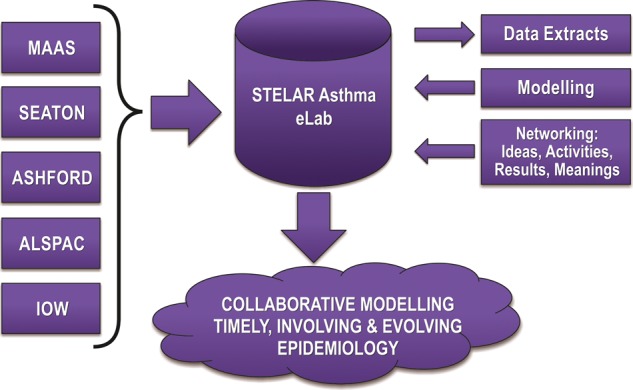
Study Team for Early Life Asthma Research (STELAR) Asthma e-Lab: platform for uniform storage and interaction for UK birth cohorts data. ALSPAC, Avon Longitudinal Study of Parents and Children; ASHFORD, Ashford cohort (Kent); IOW, Isle of Wight cohort; MAAS, Manchester Asthma and Allergy Study; SEATON, Study of Eczema and Asthma To Observe the Effects of Nutrition (Aberdeen).

To carry out the analyses and apply a graphical modelling approach to this large, complex dataset, the data must be accessible to the analysts, along with consistent information about the study measurements and the assumptions underlying the relations reflected in the annotated dependency graphs representing the problem space of asthma. To achieve this, we created a secure web-based research environment (*Asthma e-Lab*; http://www.asthmaelab.org) to support consistent recording, description and sharing of data, computational statistical methods and emerging findings across the cohorts. The Asthma e-Lab serves as a data repository populated with a unified dataset from our well-defined birth cohorts; in addition, it provides the computational resources and a scientific social network to support timely, collaborative research across the consortium (figure 1). The activities of data managers and investigators from the five STELAR sites are visible to one another, supporting team coordination and peer support, while creating a record of activity to ensure transparency. Researchers inputting the data can see how the data are being used in the analyses and receive on-line training via the e-Lab, thereby harmonising the relevant knowledge, skills and practices needed to create a consistent STELAR dataset. Emerging findings are shared with all sites via the e-Lab, linked to explanations of analytical methods that might not be familiar to all participants, thereby creating a scientific social network enriching the ongoing modelling and interpretation.

#### Mapping the study variables across the cohorts

Data managers from each cohort have mapped and annotated the recorded variables across the studies consistently. This mapping necessitates detailed descriptions (metadata) for each variable. Details held on case report forms in all five cohorts are quality assured through an online presubmission process before incorporation into the research-ready dataset.

#### Managing data securely

The privacy of participants is fully protected through the use of fine-grained access control and removal of identifiable data items, at the same time as opening up the analysis process to a range of investigators across the project.

#### Managing research questions and performing analyses

The Asthma e-Lab assembles the relevant components of research for each question in a ‘Research Object’, containing data extracts, research protocols, statistical or machine learning codes, annotated result logs, manuscripts and others. The code behind each analysis is documented and linked to the data and metadata for each variable involved.

#### Networking around results

Investigators can comment on results in near real time via the e-Lab, facilitating timely discussion over the modelling. This, in turn, leads to documented refinements of model structure. In addition, the chains of collective thought behind the interpretations of findings are captured. An online STELAR community is nurtured to maximise the transfer of knowledge between different areas of expertise. Some researchers are more familiar than others with the analytical methods or pathophysiological mechanisms around specific questions. The ‘scientific social network of analysis’ created by the e-Lab maximises the potential insights that can be generated from the consortium as a whole by enabling knowledge transfer that allows researchers to access expertise invested in groups other than their own.

#### Audit trail

We preserve the raw data and the metadata required to reproduce each analysis. All discrete investigations (published and unpublished) are thus easy to find, share and reuse as ‘Research Objects’, building on leading research into scientific knowledge management.^4^ Our approach ensures that the methodologists (statisticians, mathematicians and computer scientists) and applied researchers (clinicians and basic scientists) share consistent information about the data and methods used in specific analyses. Analyses can easily be reproduced and shared between investigators, at different sites, at all stages of analysis, since the assumptions behind each analysis are recorded in a structured way alongside the scripts that execute the calculations. In this way, the data will be preserved in the contexts of the analyses, making them available and accessible for use by external researchers via Asthma e-Lab.

#### Access to e-Lab

The e-Lab engineers work with the Steering Committee to enable different levels of sharing of data and analyses, ensuring that the e-Lab technical infrastructure reflects the agreed governance process. The future releases of the e-Lab will include additional functionality and capacity for inclusion of other cohorts.

## The interdisciplinary dialogue

The construction and running of this secure web-based resource for managing the data, metadata and collaborative analysis will encourage and facilitate the iterative interdisciplinary dialogue between clinicians, statisticians, computer scientists, mathematicians, geneticists and basic scientists, all working on a common problem,^5^ a 21st-century ‘Team Science’. The successful completion of such a programme may enable us to determine true asthma endotypes, to identify endotype-specific environmental protective and susceptibility factors, and then for each asthma endotype to discover specific biomarkers and identify target pathways and molecules for drug discovery.
